# Dauciform roots affect the position of the neighboring plants on the economic spectrum in degraded alpine meadows

**DOI:** 10.3389/fpls.2023.1277013

**Published:** 2023-10-23

**Authors:** Rong Fan, Yulin Huang, Wanting Liu, Songlin Jiang, Wenli Ji

**Affiliations:** College of Landscape Architecture and Arts, Northwest A&F University, Yangling, Shaanxi, China

**Keywords:** dauciform root, neighbouring plants, degraded meadow, economic spectrum, Cyperaceae

## Abstract

**Background and aims:**

Special root structures that can dissolve insoluble phosphorus locked in soil are supposed to contribute not only to the growing status of themselves but also to the neighbouring plants. However, whether dauciform roots have any effect on the neighbouring plants and how does it respond to meadow degradation had not been studied.

**Methods:**

Alpine meadows with different degradation statuses were selected and the functional traits of *Carex filispica* and the co-occurring species *Polygonum viviparum* were measured to explore their response to degradation, as well as the response of *Polygonum viviparum* to the dauciform roots of *Carex filispica*.

**Results:**

The results showed that 1) the number of dauciform roots decreased with the intensifying degradation, positively related to available phosphorus in the soil and negatively related to the aboveground phosphorus of *Carex filispica*. 2) *Carex filispica* and *Polygonum viviparum* are similar in specific leaf area and specific root area, yet different in the phosphorus content. The available phosphorus in the soil was negatively related to the aboveground phosphorus of *Carex filispica* and positively related to that of *Polygonum viviparum*. 3) When lightly degraded, the proportion of dauciform roots had positive effects on the aboveground resource-acquiring traits of *Polygonum viviparum*, which were no longer significant at heavy degradation. 4) *Polygonum viviparum* and *Carex filispica* without dauciform roots have similar performance: a decrease of belowground carbon with the increasing degradation, and a trend toward resource conservation with the increasing proportion of dauciform roots, which did not exist in *Carex filispica* with dauciform roots.

**Conclusion:**

Our study found that dauciform roots had a beneficial effect on the resource acquisition of their neighbouring plants. However, due to the uncontrollable nature of natural habitats, whether this effect is stable and strong enough to be performed in ecological restoration requires further lab-controlled studies.

## 1 Introduction

Phosphorus (P), an essential massive element required for plant growth, is abundant in natural soil, yet mostly as insoluble P and hardly available for plants (Richardson et al., 2009). P was found to be a restrictive factor for plant growth in meadows (Fridley, 2002), especially at high altitudes, which are generally characterized by severe nitrogen (N) deposition which intensifies the P limitation (Wright et al., 2004; Craine and Jackson, 2010; Penuelas et al., 2013; Fan et al., 2020). With the intensifying P limitation, traits that contribute to P uptake are increasingly associated with the performance and existence of plants (Turner, 2008; Olde Venterink, 2011). Thus, different morphological and physiological variations in roots have occurred to enhance P-acquisition (Lambers et al., 2006): most plants go for mycorrhizae, but a few plants develop specific root structures such as cluster roots and dauciform roots (Brundrett, 2009; Raven et al., 2018).

Dauciform root (DR), with a swollen axis and dense long root hairs, is mostly formed by Cyperaceae under P deficiency (Da Vies et al., 1973; Shane et al., 2006). In severely P-impoverished habitats such as south-western Australia, DR-forming plants have a high diversity (Shane et al., 2005), yet in the natural habitat of China, they are only described in degraded alpine meadows in the northwest of Yunnan Province (Gao and Yang, 2010). As we know, the available P is increasingly limited with the degradation of alpine meadows (Fan et al., 2020). So, is the formation of DRs related to the limited P in these degraded meadows? And does the number of dauciform roots increase with the increasing degradation and the decreasing available P?

Interesting fact, in these degraded alpine meadows, two dominant species stably occur together, *Carex filispica*, a species of Cyperaceae found to form dauciform roots here, and *Polygonum viviparum* (Gao and Yang, 2010). Species that can survive are often the ones with life strategies to adapt to the local environments (Grime, 2006), which can be reflected and quantified by their functional traits (McIntyre et al., 1999; Reich, 2014). These traits can be better predicted collaboratively, rather than independently (Watson and Szathmáry, 2016; Sporbert et al., 2021). The leaf economic spectrum (LES) indicates that these co-variant traits represent a trade-off between construction cost and longevity: they either have higher specific leaf area, photosynthetic capacity, respiration, leaf N, P content, and a shorter lifespan, or the other way around (Wright et al., 2004; Wright et al., 2007; Suding et al., 2008; Reich, 2014). Similarly, the root economic spectrum (RES) indicates that generally, roots with higher specific root area and N, P content tend to be more resource-acquiring yet short-lived, and vice versa, although it may be complicated by special root structure (Freschet et al., 2010; Carmona et al., 2021). So, the very existence of *C. filispica* and *P. viviparum* in alpine meadows indicates that they both have functional traits to acquire resources and survive the environments, the question is, of these traits, what do they have in common and how do they differ?

Furthermore, the effect of neighbouring plants can complicate the response of plants to environments. Functioning similarly to cluster roots (Shane et al., 2006), dauciform roots can dissolve insoluble P with their exudates, thereby increasing available P in the soil (Shane et al., 2006). Such dissolution of P is supposed to be beneficial not only for the growing status of the plant itself but even for the neighbouring plants (Muler et al., 2014), which has already been proved on cluster roots (Gardner and Boundy, 1983; Horst and Waschkies, 1987; Cu et al., 2005), while the relationship between dauciform roots and the neighbouring plants has never been addressed. However, this beneficial effect of cluster roots on neighbouring plants is expected to be weakened in ecosystems with greater competition (Callaway, 2007), while the competition for available soil nutrients, especially P, is found to increase with the increasing meadow degradation (Niu et al., 2016). So, can *P. viviparum* benefit from the dauciform roots of *C. filispica*, and is this beneficial effect weakened with the increase of degradation?

To address the above questions, we proposed these hypotheses: (a) the number of dauciform roots increases with the increasing degradation and the decreasing available P in the soil. (b) *P. viviparum* and *C. filispica* have similar resource-acquisitive traits in response to the degradation. (c) the growth status of *P. viviparum* has a positive correlation with the amount of dauciform roots of *C. filispica*, which weakens with the increasing degradation. Eight alpine meadow plots with two stages of degradation were selected in Baima Snow Mountain and the properties of dauciform roots, as well as the LES and RES traits of *C. filispica* and *P. viviparum*, were measured. The role of dauciform roots on neighbouring species was explored to provide a reference for future studies and the potential value of ecological restoration of Cyperaceae.

## 2 Methods

### 2.1 Study area

The study area is in Baima Snow Mountain National Nature Reserve, Yunnan Province, with a mean annual temperature of -1.0°C and precipitation of 600-650mm (Gao and Yang, 2016). The site selected is located at Baima Snow Mountain Pass at an altitude of 4300m (28°20’9’’N, 99°4’36’’E), which is exposed to a certain degree of human disturbance due to its closeness to the road. The area is mainly an alpine scrub-meadow zone, dominated by *Carex filispica* and *Polygonum viviparum*, and contains *Eleocharis yokoscensis*, *Gentiana scabra*, and *Veronica didyma*, et al.

### 2.2 Measurements of soil properties and functional traits

Our experiments were performed in September 2021 and 2022. In September 2021, using coverage as an initial reference for degradation, eight plots were selected and measured for their soil physical and chemical properties, based on which the plots were then classified into light degradation (n=4) and heavy degradation (n=4) by Cluster analysis. Meanwhile, the functional traits related to the economic spectrum were measured for *C. filispica* and *P. viviparum*: Specific leaf area (SLA), aboveground phosphorus (AGP), photosynthetic capacity (Pn_max_), specific root area (SRA), and belowground phosphorus (BGP) were selected because they are key components of LES and RES, which reflect the trade-off between resource-acquisition and self-defense (Wright et al., 2004; Poorter et al., 2010; Reich, 2014), while leaf area (LA), aboveground carbon (AGC), root surface area (RA), and belowground carbon (BGC) were selected to reflect the accumulation and allocation of carbon gain (Hermans et al., 2006). However, *C. filispica* in the 2021 experiment was measured as a whole, which was then found to be divided into individuals with and without DRs. Therefore, follow-up experiments were performed in September 2022, in which the above functional traits of individuals with and without DRs were measured respectively.

Soil properties: Each plot was measured three times by an SC-900 soil compactness meter for the average value of soil compaction (SC) between the depth of 0-20 cm, and the ring knife soil samples were taken at the depth of 0-10cm and 10-20cm to calculate the bulk density (BD). Soil samples were taken to measure soil chemical properties such as organic carbon (OC), total phosphorus (TP), available phosphorus (AP), total nitrogen (TN), and available nitrogen (AN). OC was determined by colorimetry after oxidation with a mixture of K_2_Cr_2_O_7_ and H_2_SO_4_ (Bao, 2000), TP was determined by the vanado-molybdate method (Horwitz, 1975) after digestion with H_2_SO_4_-HClO_4_, AP was measured using a UV-vis spectrophotometer by the methods of Bao (Bao, 2000), TN was determined using the Kjeldahl acid-digestion method with an auto-analyzer, and AN was measured by the Alkaline diffusion methods of Bao (Bao, 2000).

Functional traits: the chosen functional traits were measured for *C. filispica* and *P. viviparum*, with three replicates for each species in each plot. All the leaves from the three individuals were removed and measured by LI-COR portable leaf area meter for the average leaf area. Their root was scattered in the water and scanned by an LA-S root analyzer for the root surface area. The SLA (leaf area/leaf dry weight) and SRA (root surface area/root dry weight) were then calculated. The Pn_max_ was calculated from the light carves recorded by the LAI-6400 photosynthesis system, with the temperature set at 25°C. The carbon (C) and phosphorus (P) contents were measured separately for their aboveground and belowground parts. Carbon was determined by colorimetry after oxidation with a mixture of K_2_Cr_2_O_7_ and H_2_SO_4_ (Bao, 2000), and phosphorus was determined by the Vanadium molybdate yellow colorimetric method after digestion with H_2_SO_4_-H_2_O_2_ (Bao, 2000). We picked random individuals of *C. filispica* and observed their whole root system using an XTL-3B stereoscopic microscope, until we collected 10 individuals with DRs for each plot and recorded the number of DRs (lateral roots with swollen axes) per plant. The proportions of *C. filispica* with DRs were recorded, and their absolute cover of each plot was calculated (C.f with DRs proportion= the total cover of *C. filispica* × the proportion of individuals with DRs).

### 2.3 Statistical analyses

The analyses were performed using SPSS (ver 19.0, IBM, Armonk, NY, USA) and the figures were produced using Origin (ver 2022, OriginLab, USA). One-way ANOVAs were used to test for differences in each functional trait of *C. filispica* and *P. viviparum* at different degradation, and *post-hoc* tests were tested by LSD if there were differences. General linear regression models (GLM) were used to simulate the correlation between the functional traits and the environmental factors. In addition to the linear terms, models were fitted with quadratic terms for the functional traits and the proportion of dauciform roots, to test for the non-linear effects. Pearson correlation analysis was used to test the correlation among the DR properties, the functional traits, and the environmental factors. Principal component analysis (PCA) was used to downscale the standardized functional traits related to the economic spectrum of leaves and roots and to calculate the PC1 value. The structural equation models were analyzed by Amos 21.0 (Amos Development Co., Armonk, NY, USA) to figure out the effects of different factors on the values of the first axis of each species.

## 3 Results

The soil chemical and physical properties of the light-degraded and heavy-degraded plots were shown in **Table 1**. Apart from the available P, all the nutrients were significantly different between the two stages of degradation, as well as the physical properties, yet the composition of the community showed no significant difference (**Table 1**).

**Table 1:** Comparison of the environments at different degradation.

	Light degradation	Heavy degradation	*p*
OC(mg/g)	69.33 ± 5.49	37.21 ± 1.76	0.001^**^
TP(mg/kg)	1158.25 ± 69.20	829.85 ± 41.53	0.007^**^
AP(mg/kg)	11.43 ± 3.41	3.63 ± 0.97	0.070
TN(mg/kg)	4690.00 ± 214.13	2810.00 ± 77.46	0.000^***^
AN(mg/kg)	614.75 ± 24.90	319.00 ± 23.78	0.000^***^
SC(Pa)	1459.85 ± 56.86	1701.73 ± 62.72	0.029^*^
BD(g cm^-1^)	1.05 ± 0.08	1.31 ± 0.07	0.042^*^
C.f cover	27.25 ± 6.89	19.75 ± 2.02	0.336
P.v cover	28.75 ± 4.87	24.50 ± 4.19	0.533

Values represent Means ± SE (standard errors).

OC, organic carbon; TP, total phosphorus; AP, available phosphorus; TN, total nitrogen; AN, available nitrogen; SC, soil compaction; BD, bulk density. C.f cover, the absolute cover of Carex filispica; P.v cover, the absolute cover of Polygonum viviparum. ^*^ p<0.05; ^**^ p<0.01; ^***^ p<0.001.

As shown in **Table 2**, most of the differences were interspecific ones between *C. filispica* and *P. viviparum*: whether degraded or not, *C. filispica* had significantly lower P and higher C than *P. viviparum*, both aboveground and belowground. *P. viviparum* had significantly higher leaf area and root surface area than *C. filispica*, as well as the photosynthetic capacity, although, their specific leaf area and specific root area showed no significant difference.

**Table 2:** Comparison of functional traits of *Carex filispica* (C.f) and *Polygonum viviparum* (P.v) at different degradation.

	C.f in LD	C.f in HD	P.v in LD	P.v in HD	*p*
AGC(mg/g)	491.81 ± 7.67a	477.52 ± 8.10ab	462.58 ± 8.66b	426.11 ± 3.95c	0.033^*^
BGC(mg/g)	490.36 ± 5.74a	474.68 ± 8.17a	427.35 ± 4.16b	402.08 ± 3.89c	0.000^***^
AGP(mg/g)	3.06 ± 0.10b	3.16 ± 0.03b	4.39 ± 0.23a	3.96 ± 0.20a	0.025^*^
BGP(mg/g)	2.98 ± 0.05b	2.93 ± 0.04b	3.80 ± 0.08a	3.80 ± 0.14a	0.003^**^
Pn_max_(µmolCO_2_ m^-2^ s^-1^)	4.87 ± 1.06b	3.01 ± 0.64b	8.17 ± 0.81a	7.63 ± 1.35ab	0.011^*^
LA(cm^2^)	0.32 ± 0.07b	0.63 ± 0.11b	2.54 ± 0.23a	2.63 ± 0.36a	0.000^***^
SLA(cm^2^ g^-1^)	243.07 ± 29.61a	268.39 ± 30.27a	227.95 ± 10.92a	200.52 ± 19.49a	0.289
RA(cm^2^)	6.46 ± 1.22b	7.26 ± 1.06b	35.34 ± 5.94a	36.08 ± 8.93a	0.006^**^
SRA(cm^2^ g^-1^)	92.12 ± 6.83a	132.47 ± 17.13a	187.38 ± 80.24a	218.20 ± 123.3a	0.642

Values represent Means ± SE (standard errors). LD, light degradation; HD, heavy degradation; AGC, aboveground carbon; BGC, belowground carbon; AGP, aboveground phosphorus; BGP, belowground phosphorus; Pn_max_, photosynthetic capacity; LA, leaf area; SLA, specific leaf area; RA, root surface area; SRA, specific root area. ^*^ p<0.05; ^**^ p<0.01; ^***^ p<0.001.

**Figure 1:**
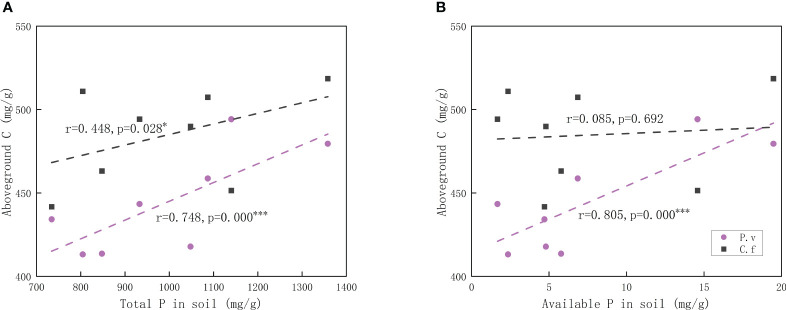
Relations of **(A)** aboveground carbon (C) in plants and total phosphorus (P) in soil; **(B)** aboveground carbon in plants and available phosphorus in soil. Regression lines are taken from phylogenetic generalized least squares models. The black dashed lines indicate Carex filispica (C.f), purple dashed lines for Polygonum viviparum (P.v). Asterisks indicate significant correlations, ^*^ *p*<0.05; ^***^ *p*<0.001.

As can be seen from **Table 2**, the only difference in the response to degradation between *C. filispica* and *P. viviparum* was that as the degradation increased, there was a significant decrease in carbon concentration both above- and belowground in *P. viviparum*, while not significant in *C. filispica*. **Figure 1** showed that aboveground C of *P. viviparum* was positively related to both total and available P in soil, while *C. filispica* was only positively related to total P and unaffected by available P.

Further analysis (**Table 3**) showed that the belowground C of *C. filispica* without DRs decreased with the increasing degradation, just like *P. viviparum*, yet *C. filispica* with DRs showed no such trend.

**Table 3:** Comparison of carbon concentration of *Carex filispica* (C.f) with and without dauciform roots (DRs) and *Polygonum viviparum* (P.v) at different degradation.

	P.v in LD	P.v in HD	C.f with DRs in LD	C.f with DRs in HD	C.f without DRs in LD	C.f without DRs in HD	*p*
AGC (mg/g)	462.58 ± 8.66c	426.11 ± 3.95d	479.25 ± 9.86bc	465.14 ± 12.06c	504.38 ± 7.20a	489.91 ± 5.47ab	0.000^***^
BGC (mg/g)	427.35 ± 4.16c	402.08 ± 3.89d	480.89 ± 8.11ab	477.83 ± 13.49b	499.83 ± 4.69a	471.54 ± 4.79b	0.000^***^

Values represent Means ± SE (standard errors). LD, light degradation; HD, heavy degradation; AGC, aboveground carbon; BGC, belowground carbon. ^***^ p<0.001.

**Figure 2:**
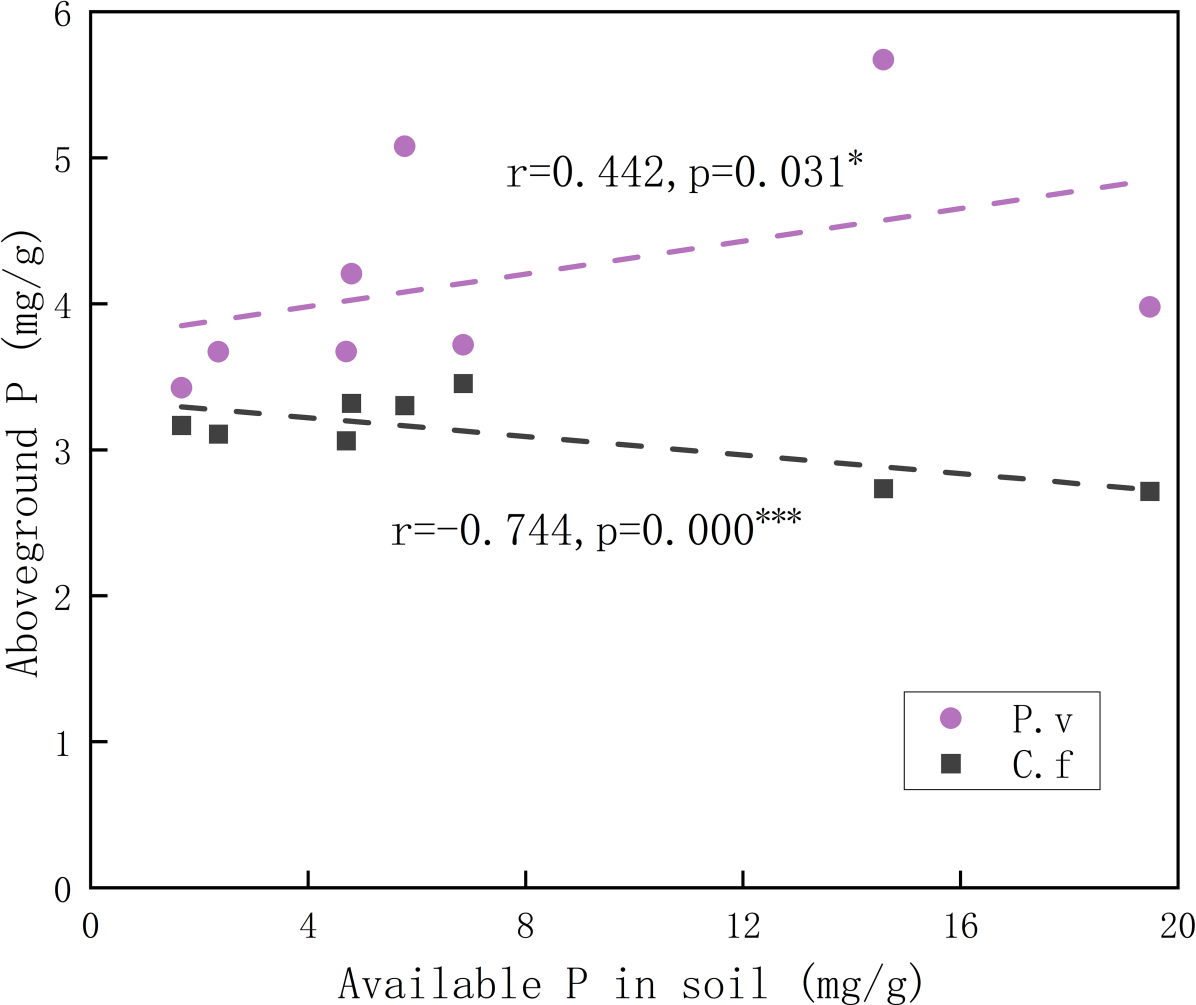
Relations of aboveground phosphorus (P) in plants and available phosphorus in soil. Regression lines are taken from phylogenetic generalized least squares models. The black dashed line indicates Carex filispica (C.f), purple dashed line for Polygonum viviparum (P.v). Asterisks indicate significant correlations, ^*^ *p*<0.05; ^***^ *p*<0.001.

**Figure 3:**
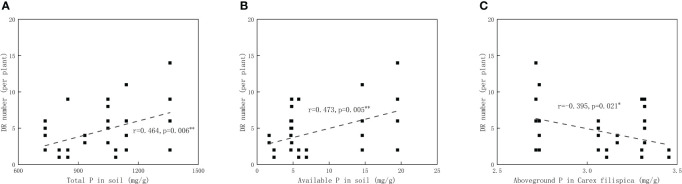
Relations of **(A)** the average number of dauciform roots (DR) and total phosphorus (P) in soil; **(B)** the average number of dauciform roots and available phosphorus in soil. **(C)** the average number of dauciform roots and aboveground phosphorus in *Carex filispica*. Regression lines are taken from phylogenetic generalized least squares models. Asterisks indicate significant correlations, ^*^ *p*<0.05; ^**^ *p*<0.01.

Available P in soil was positively related to aboveground P in *P. viviparum*, yet negatively related to that in *C. filispica* (**Figure 2**). The average number of dauciform roots significantly decreased with the increase in degradation (**Table 4**). The average number of dauciform roots was positively related to total P and available P in the soil, yet negatively related to the aboveground P in *C. filispica* (**Figure 3**).

**Table 4:** Comparison of properties of dauciform roots (DR) at different degradation.

	Light degradation	Heavy degradation	*p*
DR number(per plant)	5.720 ± 0.859	3.190 ± 0.526	0.020^*^
DR proportion(%)	49.0 ± 9.7	58.8 ± 16.4	0.625
C.f with DR proportion (%)	16.922 ± 4.120	18.152 ± 4.546	0.848

DR number, the average number of dauciform roots per plant; DR proportion, the proportion of individuals with dauciform roots; C.f with DR proportion, the absolute cover of Carex filispica with dauciform roots. Values represent Means ± SE (standard errors). ^*^ p<0.05.

**Figure 4:**
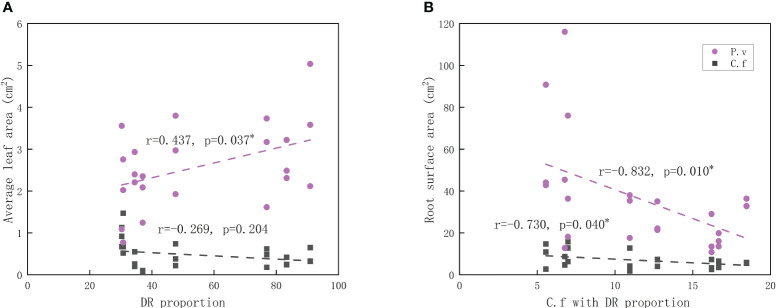
Relations of **(A)** average leaf area and the proportion of dauciform roots (DR); **(B)** root surface area and the cover of *Carex filispica* with dauciform roots. The black dashed lines indicate *Carex filispica* (C.f), purple dashed lines for *Polygonum viviparum* (P.v). Asterisks indicate significant correlations, ^*^ *p*<0.05.

GEM showed that the proportion of dauciform roots was positively related to the leaf area of *P. viviparum* and showed no such relation with *C. filispica*. On the other hand, the relative cover of *C. filispica* with dauciform roots showed negative relations with the root surface area of both *C. filispica* and *P. viviparum* (**Figure 4**).

**Figure 5:**
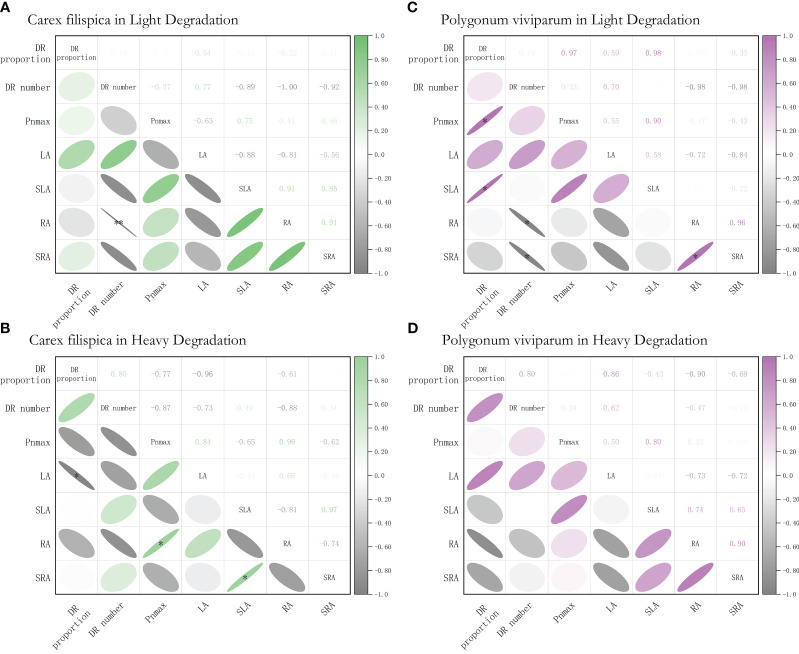
Relationships among dauciform root (DR) traits, and the functional traits of *Carex filispica* and *Polygonum viviparum* at different degradation, described by Pearson correlation coefficients. **(A)** relationships of *Carex filispica* at light degradation. **(B)** relationships of *Carex filispica* at heavy degradation. **(C)** relationships of *Polygonum viviparum* at light degradation. **(D)** relationships of *Polygonum viviparum* at heavy degradation. Pn_max_, photosynthetic capacity; LA, leaf area; SLA, specific leaf area; RA, root surface area; SRA, specific root area. Asterisks indicate significant correlations, ^*^ *p*<0.05, ^**^ *p*<0.01.

Further analysis of *P. viviparum* found that at light degradation, the proportion of dauciform roots had a positive relation with their photosynthetic capacity and specific leaf area, while the number of dauciform roots showed a negative relation with the root surface area and specific root area. Yet, all these correlations between DR properties and their functional traits were not significant at heavy degradation (**Figure 5**).

Principal component analysis (PCA) of the economic spectrum traits of *C. filispica* and *P. viviparum* was performed. **Table 5** showed that a higher PC1 value indicates a tendency of higher SRA, Pn_max_, BGP, SLA, and AGP, which means that the plants are leaning toward the resource-acquisitive side on the plant economic spectrum. The PC1 value of *P. viviparum* was negatively correlated with the relative cover of *C. filispica* with dauciform roots (*r*=-0.749, *p*=0.032).

**Table 5:** Trait loadings and eigenvalues of the economic spectrum trait variation explained by successive principal components (PC) in the trait PCA.

	PC1	PC2
SRA	0.773	-0.183
Pn_max_	0.731	0.196
BGP	0.610	0.427
SLA	0.059	-0.837
AGP	0.166	0.630

SRA, specific root area; Pn_max_, photosynthetic capacity; BGP, belowground phosphorus; SLA, specific leaf area; AGP, aboveground phosphorus.

**Figure 6:**
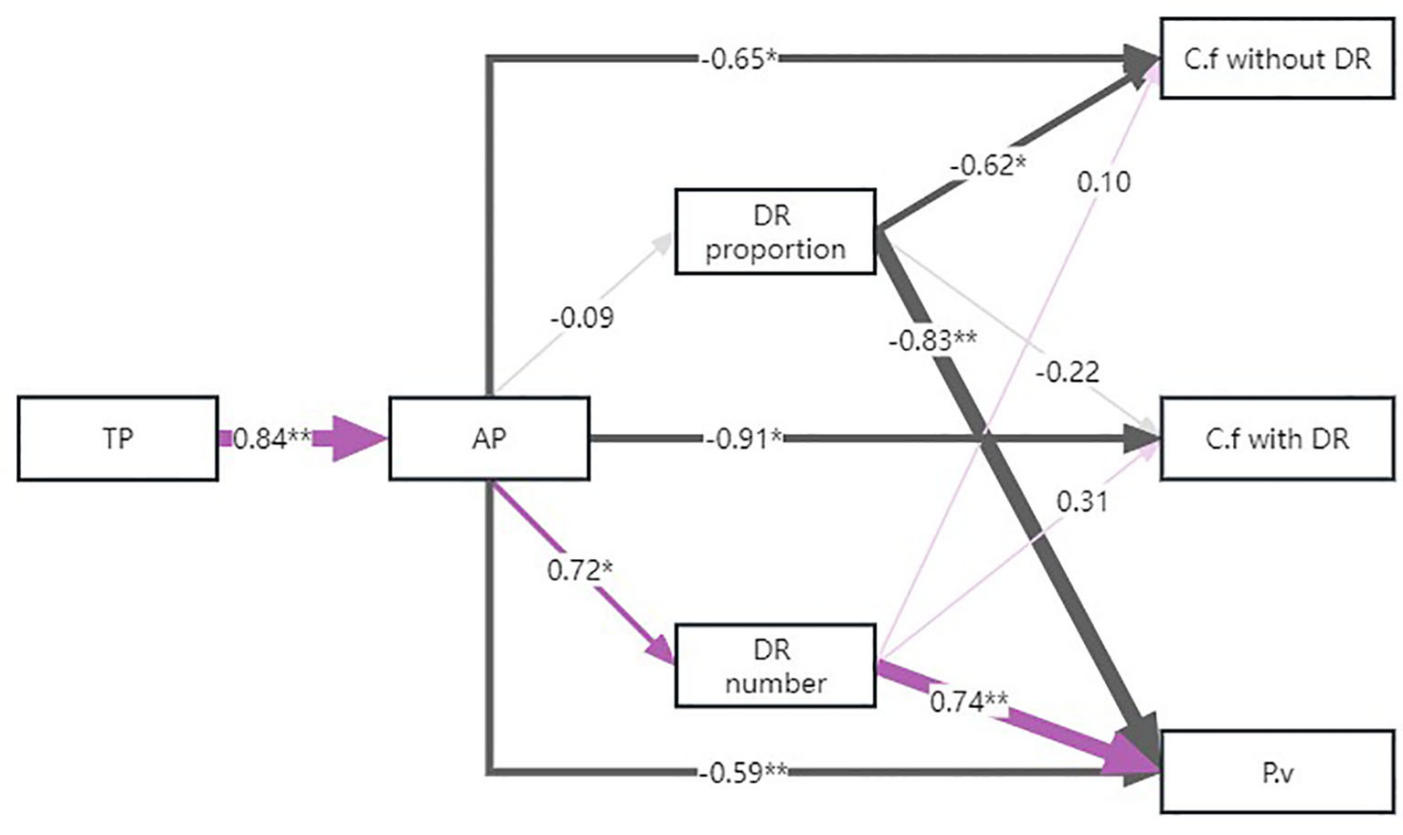
The structural equation model of effects of soil phosphorus (P) and dauciform root (DR) properties on the PC1 value of the economic spectrum of *Carex filispica* (C.f) with and without dauciform roots and *Polygonum viviparum* (P.v). Purple lines represent positive impacts while dark grey lines represent negative ones. Lines of different thickness and the numbers represent the size of the standardized path coefficients, and asterisks represent the level of significance (^*^ *p*<0.05, ^**^ *p*<0.01).

Structural equation modeling showed that available P in the soil had a negative relation with the PC1 value of the economic spectrum of each group (**Figure 6**). However, the proportion of *C. filispica* with dauciform roots only had negative effects on the value of *C. filispica* without dauciform roots and *P. viviparum*, leading them towards the conservative side, while this relationship was not significant on *C. filispica* with dauciform roots themselves.

## 4 Discussion

### 4.1 The number of dauciform roots decreases with the increasing degradation, regulated by the aboveground P of *C. filispica*

Alpine meadow ecosystems are generally characterized by an abundance of total nutrients and a shortage of available nutrients (Fan et al., 2020). At different stages of degradation, the functional traits of plants are limited by different soil nutrients (Li et al., 2016), and the competition for available P was proved to be enhanced with the increasing degradation (Niu et al., 2016). In our study, the aboveground P of *C. filispica* showed a negative relation to the available soil P while that of *P. viviparum* was positively related (**Figure 2**). This indicates that *P. viviparum* has a stronger capability of available P acquisition, which therefore disadvantages the P acquisition of *C. filispica* when there is adequate available P in the soil. In addition, a portion of *C. filispica* had produced dauciform roots, which could provide a physiological advantage in P acquisition in the lack of available P by dissolving and utilizing the P fixed in the undecomposed organic matter of soil (Shane et al., 2005; Playsted et al., 2006; Masuda et al., 2020), therefore explains the inverse ratio their aboveground P showed with soil available P (**Figure 2**).

Despite that dauciform roots are considered an adaptation to low P (Lambers et al., 2006; Oliveira et al., 2015), species with dauciform roots are widely distributed worldwide from low to high P (Shane et al., 2005; Zemunik et al., 2015; Gusewell and Schroth, 2017). Our study area is not a typical P-deficient environment, therefore, only a portion of *C. filispica* produced dauciform roots, and only in small amounts (Gao and Yang, 2016). Since aboveground P in *C. filispica* is negatively related to available soil P (**Figure 2**), the average number of dauciform roots, while negatively related to aboveground P in *C. filispica*, turned out to be positively related to available soil P (**Figure 3**) and decreased with the increasing degradation (**Table 4**). Being contrast to our hypothesis, our result is consistent with previous studies that the formation of dauciform roots is regulated by P in the aboveground parts of plants, not in the soil (Shane et al., 2006).

### 4.2 *C. filispica* and *P. viviparum* are different in the C and P content, yet similar in SLA and SRA

Species that can survive are often the ones with ‘suitable’ traits to adapt to the local environments (Grime, 2006). The variation in functional traits can be mainly explained by edaphic factors (Salazar Zarzosa et al., 2021), and the variation in root economic traits was proved to drive aboveground biodiversity effects (Bu et al., 2017). In our study, the resource-acquisitive traits of *C. filispica* and *P. viviparum* were similar while the accumulation of carbon gain was significantly different: their SLA and SRA appeared to be of no significant difference, even though *P. viviparum* has a significantly higher leaf area and root area than *C. filispica*. Interspecifically, the above- and belowground P of *C. filispica* was significantly lower than that of *P. viviparum*, and their above- and belowground C was higher, regardless of the stage of degradation (**Table 2**). In a previous study, a typical non-mycorrhizal carboxylate-releasing species in Cyperaceae showed relatively lower Leaf P content compared to other species (Zhou et al., 2022), which is consistent with our result. As for their response to degradation, there is only a significant difference in above- and belowground C content: *P. viviparum* decreased as the degradation intensified, while those of *C. filispica* remained stable (**Table 2**). As mentioned before, *P. viviparum* appears to have stronger competitiveness for P, while the dauciform roots of *C. filispica* can stabilize their P content under P-impoverished environments. It may also explain the fact that the aboveground C of *C. filispica* only showed a positive correlation with total soil P while *P. viviparum* showed relations with both total P and available P (**Figure 1**): *C. filispica* is not dependent on available P because of the presence of dauciform roots, and might even be limited under high available P environments owing to the competition from *P. viviparum*.

Compared to the interspecific variability of traits, intraspecific ones were generally considered negligible, however, they were recently proven to have significant effects on the community and ecosystem functioning (Fu et al., 2020). The filtering of environments is not only interspecific but also intraspecific, that is, if the environment is eliminating species with lower leaf carbon content, it is most likely that individuals with lower carbon content within the species will also be eliminated (Sandel et al., 2021). The carbon content in *P. viviparum* was found to decrease significantly with the increasing degradation, while that of *C. filispica* showed no significant difference (**Table 2**). After being divided into two groups according to the presence of dauciform roots, the belowground C of *C. filispica* without dauciform roots showed a significant decrease with degradation, just like *P. viviparum*, while the ones with dauciform roots remained unchanged (**Table 3**). Therefore, it can be speculated that the previous results are due to dauciform roots adapting to heavily degraded environments, thus affecting the carbon content of *C. filispica* as a whole.

### 4.3 *P. viviparum* benefits from the dauciform roots of *C. filispica*, which gets weakened with the increase in degradation

While studies on dauciform roots are extremely limited, these special root structures with the function of P-dissolving are supposed and proved to be beneficial not only for the plant itself but even for the growing status of the neighbouring plants (Muler et al., 2014). The promoting effect of cluster roots on neighbouring plants had been proven in several studies (Gardner and Boundy, 1983; Horst and Waschkies, 1987; Cu et al., 2005), however, this beneficial effect is not always reflected in P (Muler et al., 2014): in some previous studies, the neighbouring plants had both increased P and growth when planted with cluster root forming plants (Gardner and Boundy, 1983), in others, there appeared to be no difference in P while their growth was enhanced (Muler et al., 2014). The allocation of plants is usually biased towards the organs in charge of absorbing the most limited resources (Bloom et al., 1985; Poorter et al., 2012): roots are responsible for the uptake of nutrients and water while leaves are essential for photosynthesis (Rehling et al., 2021). In this study, the proportion of dauciform roots was negatively related to the root surface area of *P. viviparum* and positively related to the leaf area (**Figure 4**), which shows a relief of additional inputs to the belowground and a bias towards aboveground resources.

Furthermore, at light degradation, *P. viviparum* was affected by the proportion of dauciform roots, with increasing specific leaf area and photosynthetic capacity aboveground, decreasing root surface area and specific root area belowground (**Figure 5C**). The functional traits shown at this point can be explained by the presence of dauciform roots contributing to P-uptake, which releases the inputs for the nutrient-taking fine roots and invests in the aboveground competition for light. On the other hand, plants lacking phosphorus tend to show a decrease in stomatal conductivity (Thomas et al., 2006) and an increase in leaf dry mass per unit area (Fyllas et al., 2009; Turnbull et al., 2016). Our experiment shows that at light degradation, the lower the proportion of dauciform roots, the lower the photosynthetic rate and specific leaf area of *P. viviparum*, which shows P deficiency and can be easily explained by the P-uptaking promotion of dauciform roots. Interestingly, when the degradation increased, the functional traits of *P. viviparum* stopped being correlated with the proportion or number of dauciform roots (**Figure 5D**). In previous studies, the beneficial effect of cluster roots on other plants was expected to be weakened with greater physical stress or competition (Callaway, 2007), which is consistent with our results on dauciform roots: with the increasing degradation, dauciform roots of *C. filispica* no longer significantly contribute to the functional traits of *P. viviparum*, and the C content of *P. viviparum* suffered a great decrease.

### 4.4 The role of dauciform roots

Other than responding to the environments, functional traits can also play important roles in determining ecosystem functioning: they can be divided into response traits and effect traits, response ones describe the response of plants to the environment while effect ones describe the effect of plants on the ecosystem (Violle et al., 2007; Liu et al., 2021). A trait may correspond to one or several ecosystem functions (Sack and Buckley, 2020), while root economic traits were considered to be correlated with soil nutrient availability (Mommer and Weemstra, 2012; Freschet et al., 2021). Therefore, an alternative theory here for our result on the first hypothesis is that the dissolution of more dauciform roots increases the available P content of the soil, causing the positive correlation between the number of dauciform roots and the available soil P, also, the growth status of *P.viviparum*.

In fact, while having the same response of belowground C to the intensifying degradation, *P. viviparum* and *C. filispica* without dauciform roots also showed a similar response to dauciform roots: as the proportion of dauciform roots increased, their position on the economic spectrum was both biased in a resource-conservative direction (**Figure 6**), while individuals with dauciform roots did not have the same tendency. Our result is consistent with an earlier study showing that individuals with dauciform roots were proven to have more resource-acquiring traits after human trampling (Fan et al., 2023). Species with resource-acquisitive strategies tend to increase as disturbance intensified (Huang et al., 2021), and owing to the resource acquisition of dauciform roots contributing to the P-dissolution to ease the disturbance, all the other neighbouring plants remained resource-conservative. While intraspecific competition was mainly for belowground resources (Rehling et al., 2021), previous research showed that Cyperaceae may acquire P without depending on carboxylate release (Zhou et al., 2022). At the cost of producing dauciform roots in some of the individuals, *C. filispica* enhanced the P acquisition of the whole population, even the aboveground traits of *P. viviparum* at light degradation.

## 5 Conclusion

In this study, patterns and trends were noticed: the number of dauciform roots actually had a positive correlation with the available soil P, as well as the growth status of *P. viviparum*, which vanished with the increase of degradation. Most importantly, dauciform roots had significant effects on the position of *P. viviparum* and *C. filispica* without dauciform roots on the economic spectrum. This study contributes to the understanding of the role of dauciform roots on the companion species: with the intensifying P limitation, Cyperaceae with dauciform roots may play an important role in ecological restoration projects. However, the study area is not a typical P-deficient environment and some of the causality is still unclear due to the uncontrollable nature of the natural habitats. Whether this effect of dauciform roots is stable and strong enough to play a role in ecological restoration and subsequently be applied to the relief of phosphorus deficiency in artificial cultivation, requires further controlled variable studies.

## Data availability statement

The raw data supporting the conclusions of this article will be made available by the authors, without undue reservation.

## Author contributions

RF: Conceptualization, Formal Analysis, Investigation, Writing – original draft. YH: Investigation, Writing – review & editing. WL: Investigation, Writing – review & editing. SJ: Investigation, Writing – review & editing. WJ: Conceptualization, Funding acquisition, Supervision, Writing – review & editing.
